# Paragangliomas Arising From the Laryngeal Paraganglia: Thyroid and Laryngeal Paragangliomas With Radiology-Pathology Correlation

**DOI:** 10.7759/cureus.57613

**Published:** 2024-04-04

**Authors:** Alia Tayara, William R Townsend, Areejah Umar, Kirby G Parker, Varsha Manucha, Anne C Kane, Lana Jackson, Charlotte S Taylor

**Affiliations:** 1 Otolaryngology, University of Mississippi Medical Center, Jackson, USA; 2 Radiology, University of Mississippi Medical Center, Jackson, USA; 3 Pathology, University of Mississippi Medical Center, Jackson, USA; 4 Otolaryngology - Head and Neck Surgery, University of Mississippi Medical Center, Jackson, USA

**Keywords:** nonfunctional paraganglioma, supraglottic paraganglioma, laryngeal paraganglioma, thyroid paraganglioma, head and neck paraganglioma (hnpgl)

## Abstract

Paragangliomas are neuroendocrine tumors that arise from the embryonic neural crest cells of the extra-adrenal chromaffin and non-chromaffin cellular system. Paragangliomas arising from the laryngeal paraganglia, which occur in the thyroid and larynx, are a rare subset of paragangliomas compared to the more common locations of the carotid body, vagale, jugular, and tympanic paragangliomas. The preoperative diagnosis of both thyroid and laryngeal paragangliomas may pose a challenge due to cytological, pathological, and imaging non-specificity that overlaps with many other neoplasms. These lesions may be associated with significant intraoperative bleeding and complicated excision with adherence to nearby structures, including the recurrent laryngeal nerve. This article discusses the imaging appearance, pathological features, clinical and operative considerations and manifestations, and management of head and neck paragangliomas, as seen in two patients at our institution.

## Introduction

Paragangliomas arising from the laryngeal paraganglia, which occur in the thyroid and larynx, are a rare subset of paragangliomas compared to the more common locations of the carotid body, vagale, jugular, and tympanic paragangliomas. The laryngeal paraganglia give rise to both laryngeal and thyroid paragangliomas. Paired superior paraganglia are located in the supraglottic larynx along the superior margin of the thyroid cartilage. The vast majority of laryngeal paragangliomas are supraglottic in location, arising from the superior laryngeal paraganglia. The inferior laryngeal paraganglia are more variable in location and can be found at the level of the cricoid cartilage to the first tracheal ring and may occur within the thyroid gland [1-3]. Inferior laryngeal paraganglia may give rise to paragangliomas in the thyroid gland, subglottic larynx, or upper cervical trachea [4-6]. These lesions may be associated with significant intraoperative bleeding and complicated excision with adherence to nearby structures, including the recurrent laryngeal nerve.

The preoperative diagnosis of both thyroid and laryngeal paragangliomas may pose a challenge due to cytological, pathological, and imaging non-specificity that overlaps with many other neoplasms, particularly with limited literature available on these rare tumors. This article aims to improve the recognition of imaging and pathological features suggestive of paragangliomas to aid their management and preoperative planning.

## Case presentation

Case 1

An 80-year-old female with a history of asymptomatic thyroid enlargement diagnosed clinically as a multinodular thyroid initially presented to an outside facility with hemoptysis and was found to have abnormal circumferential vascularity of the proximal trachea. Argon coagulation of the vessels was performed, which resolved her symptoms. Eleven years later, she presented to an outside hospital after an unrelated CT cervical spine demonstrated a 5 cm right thyroid mass, prompting ultrasound that further elucidated a 5.7 × 4.6 × 4.1 cm right thyroid lobe lesion with irregular margins and likely extension beyond the capsule. Subsequent ultrasound-guided needle core biopsy was obtained, and a diagnosis of neuroendocrine tumor was rendered, supported by the positive expression for CD56, and synaptophysin with a weak expression for thyroid transcription factor 1 (TTF-1). The stains for thyroglobulin and calcitonin were negative. A differential diagnosis of medullary carcinoma, paraganglioma, and metastatic neuroendocrine tumor was suggested. T4 (1.17 ng/dL), thyroid-stimulating hormone (1.26 µlU/mL), and calcitonin (<2.0 pg/mL) levels resulted within normal ranges.

Upon transfer to our institution for further workup and treatment of thyroid malignancy, calcitonin levels were repeated, with the addition of a carcinoembryonic antigen (CEA) measurement, both within normal ranges (<5.0 pg/mL and 3.48 ng/mL, respectively). Further studies were performed with previously acquired biopsy tissue confirming negative immunostains, though the tissue positively expressed CD56 and synaptophysin. She remained admitted for additional imaging and management.

Imaging

**Figure 1:**
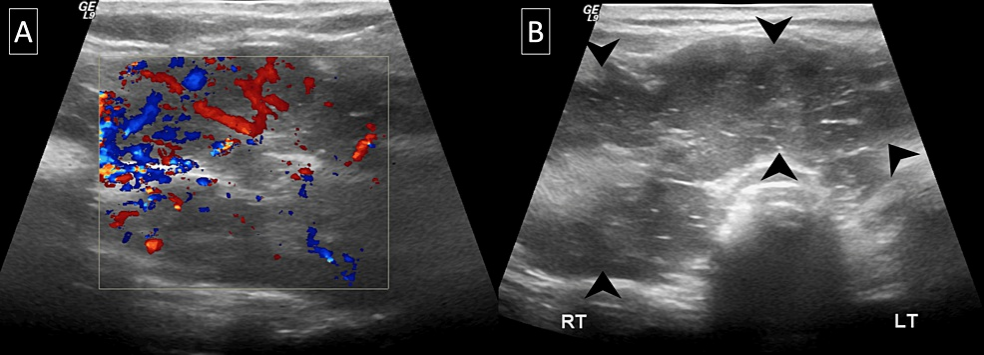
Thyroid ultrasound. Initial thyroid color Doppler (A) and grayscale (B) ultrasound images demonstrate a heterogeneous thyroid gland with an enlarged and hypervascular right thyroid lobe/isthmus (arrowheads, B).

**Figure 2:**
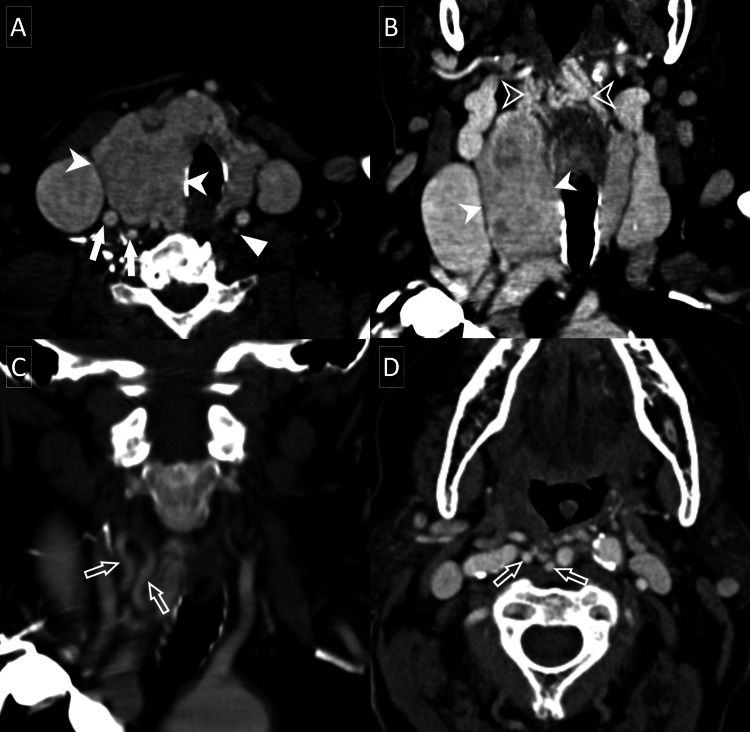
CT of the soft tissue neck with intravenous contrast axial (A, D) and coronal (B, C) views. The most recent CT of the soft tissue neck with intravenous contrast demonstrates an enlarged heterogeneously enhancing thyroid with an exophytic right thyroid lobe nodule (solid arrowheads) extending into the isthmus. The nodule is supplied by enlarged and tortuous inferior (solid arrows) and superior (hollow arrows) thyroidal arteries. Extensive arterial and venous collaterals are noted (hollow arrowheads). The normal right inferior thyroid artery is denoted by a solid triangle for comparison.

**Figure 3:**
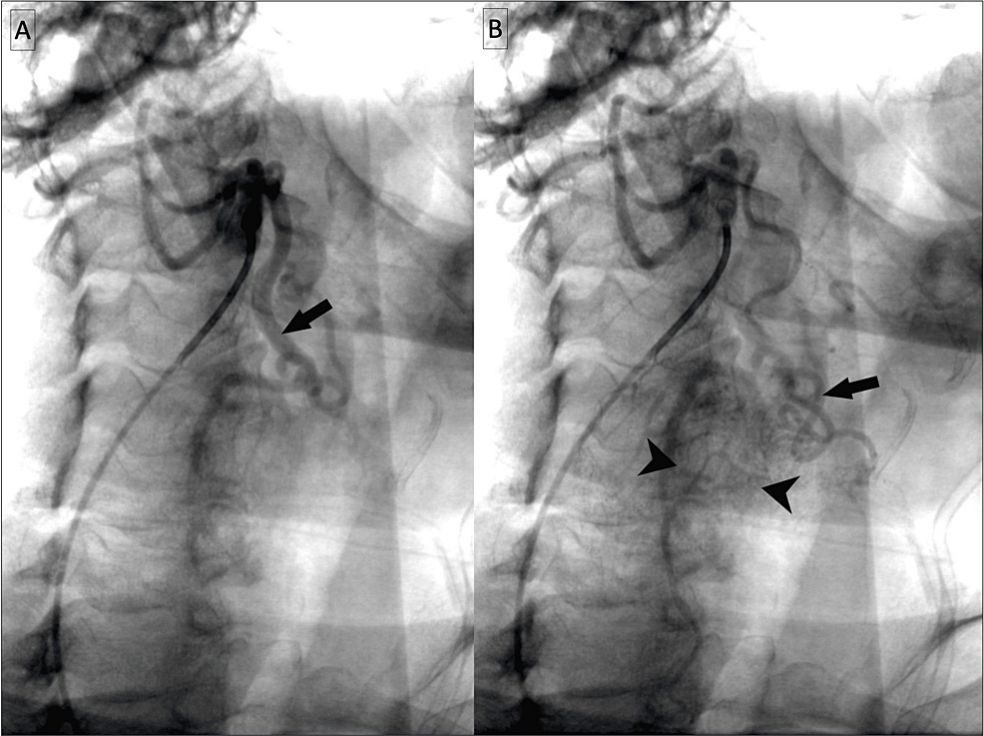
External carotid arteriogram. Early arterial (A) and late arterial (B) phases demonstrate extensive enhancement in the region of the right thyroid paraganglioma (arrowheads) supplied by a prominent superior thyroidal artery with multiple smaller collaterals (arrows).

Upon presentation to our institution, CT of the soft tissue neck with intravenous (IV) contrast demonstrated an enlarged right thyroid lobe and isthmus which was heterogeneously and avidly enhancing, unchanged in size from an outside ultrasound (US) of the thyroid (Figure 1) and a CT of the soft tissue neck from 11 years prior. Large prominent vessels were present in the retropharyngeal space, and there was an asymmetric enlargement of the right superior thyroidal artery (Figures 2, 3). On previous catheter angiography at an outside facility, these vessels were proven to have arterial supply from the superior thyroidal artery (Figure 3). No lymphadenopathy was noted.

Operative Report

The recommendation was made to proceed with a total thyroidectomy for a definitive diagnosis. The thyroid isthmus, right, and left lobes were identified using blunt dissection. All major vessels of the right thyroid were identified and addressed to maintain hemostasis. After freeing the superior right pole, the inferior right pole was released, allowing the right thyroid to be reflected medially, and the recurrent laryngeal nerve was identified and maintained. The right thyroid, once divided at the isthmus, was completely freed and sent for pathological examination. Both the superior and inferior right parathyroid glands were preserved. Considerable bleeding was encountered, and due to the high likelihood of non-aggressive pathology from stable imaging over an 11-year period, the operation was concluded with a right hemithyroidectomy.

Pathology

**Figure 4:**
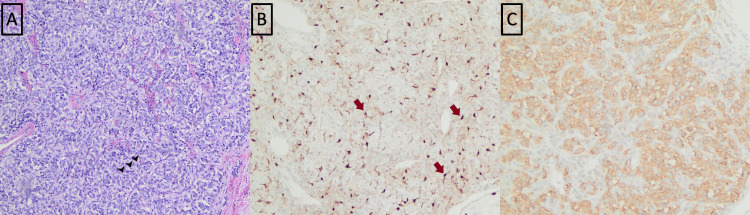
Thyroid paraganglioma microscopic evaluation. (A) Hematoxylin and eosin staining (200×). Monomorphic tumor cells arranged in a well-defined nest (zellballen) pattern surrounded by a thin fibrovascular stroma (arrowheads on a few characteristic nests). (B) S100 immunostain (200×) highlights sustentacular cells along the periphery of the cellular nests (arrows). (C) Immunostain for synaptophysin is positive in the cytoplasm of the tumor (brown pigment).

The tumor involved the entire right lobe of the thyroid and extended to the superior, anterior, and posterior surface margins, also reaching the isthmic resection margin. The tumor comprised monomorphic cells (chief cells) with round-to-oval nuclei with a fine chromatin pattern arranged in a well-defined nest (zellballen) pattern surrounded by a thin fibrovascular stroma. There was no evidence of follicular differentiation, colloid formation, mitosis, necrosis, or amyloid deposition. Immunohistochemistry revealed positive expression for synaptophysin and insulinoma-associated protein 1, with S-100 highlighting the sustentacular cells (Figure 4). Additional stains, including calcitonin, TTF-1, and CEA, were negative in the tumor cells. The stain for succinate dehydrogenase (SDHB) did not show any aberrant loss.

Case 2

A 55-year-old female presented to our institution with progressive dyspnea on exertion. She was found to have an obstructive supraglottic laryngeal mass and subsequently underwent a tracheotomy and biopsy, which showed a vascular malformation. She underwent embolization of the inferior thyroid artery and superior thyroid artery branches supplying the supraglottic mass in an attempt to shrink the mass. Subsequent examinations and imaging did not show any evidence of improvement in tumor size. Her follow-up imaging showed residual venous-predominant blood supply, and it was then recommended that she undergo microlaryngoscopy with fluoroscopy-guided sclerotherapy injections in the left supraglottic vascular malformation. As her tumor did not improve with conservative measures, it was recommended that she undergo open surgical excision of the mass with a lateral pharyngotomy approach.

Imaging

**Figure 5:**
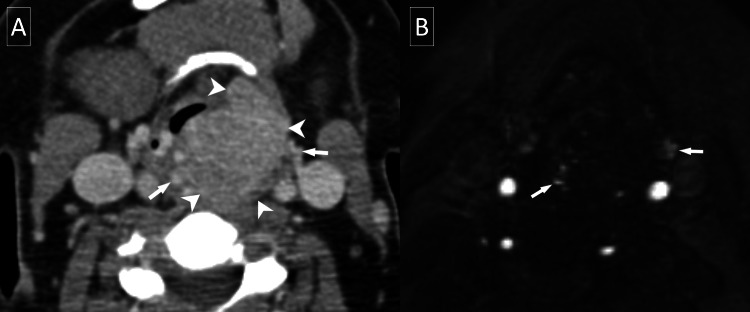
CT of the soft tissue neck with intravenous contrast. Initial axial CT of the soft tissue neck with intravenous contrast (A) demonstrates an enhancing well-circumscribed 4.7 cm mass (arrowheads) centered in the left supraglottic larynx with significant mass effect and no extralaryngeal extension. Extensive surrounding vascularity (arrows) is noted on both the CT and time-of-flight magnetic resonance angiography (B).

Initial CT of the soft tissue neck with IV contrast showed a homogenously enhancing supraglottic mass measuring up to 4.6 cm centered in the left supraglottic larynx with significant mass effect including severe narrowing of the supraglottic and glottic airway (Figure 5). Both CT and pre- and post-contrast time-of-flight magnetic resonance angiography of the neck demonstrated lateral extension of the mass into the left paraglottic and pre-epiglottic spaces, and inferior extension to the level of the true vocal cords. Prominent vessels surrounded the right lateral aspect of the mass and extended into the hypopharynx. No extralaryngeal tumor or lymphadenopathy was demonstrated.

**Figure 6:**
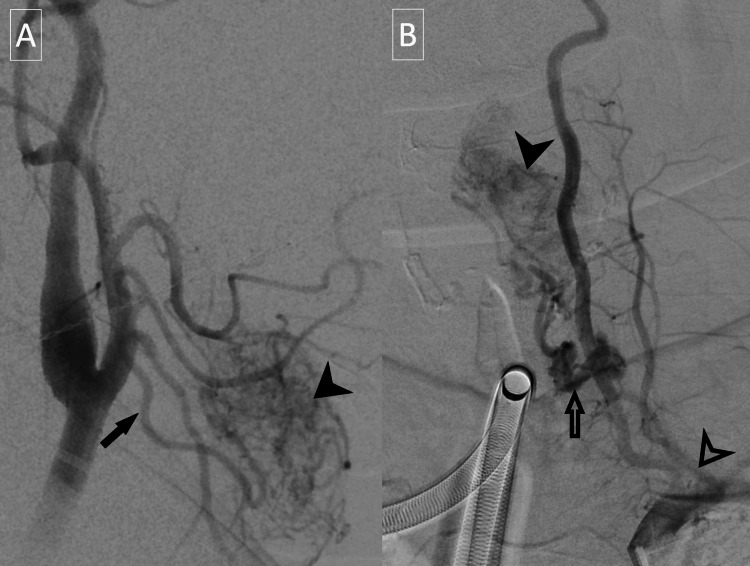
Catheter angiogram of the neck. Catheter angiogram with common carotid artery injection (A) and external carotid artery injection (B) demonstrate tumor blush of the supraglottic paraganglioma (solid arrowheads), supplied by the inferior thyroidal artery (hollow arrow) and superior thyroidal artery (solid arrow). The thyrocervical trunk is denoted by the hollow arrowhead.

A catheter angiogram of the left neck demonstrated rapid arterial blush corresponding to the supraglottic mass with extensive associated vascularity primarily supplied by the inferior and superior thyroid arteries (Figure 6).

Follow-up contrasted CT of the soft tissue neck and CT angiography neck studies over the next two years demonstrated similar size, extension, and effacement of the supraglottic airway, as well as similar prominent associated venous and arterial collaterals.

Operative Report

Direct laryngoscopy revealed a prominent left supraglottic vascular mass with no obvious involvement of the pyriform sinus. Next, the left neck incision was made, and dissection was carried down through the platysma. Superior and inferior subplatysmal flaps were then raised in standard fashion. The sternocleidomastoid muscle was unwrapped from the investing fascia. The internal jugular vein was identified and followed superiorly until the facial vein was identified. A large feeding vein connecting the facial vein to the mass was isolated and ligated. The left superior laryngeal and superior thyroid vessels were identified and ligated, with the superior laryngeal nerve isolated and protected. The lateral edge of the thyroid cartilage was identified, and the constrictor muscle was incised along the lateral border of the cartilage. The mucosa of the inner thyroid cartilage was elevated, and the vascular tumor was visualized within the paraglottic space. The mass was vascular with many feeding vessels. The mass was able to be bluntly dissected away from the inner pharyngeal mucosa layer without violation of the pharyngeal mucosa and was then sent to pathology. All feeder vessels were ligated. The constrictor muscle was sutured back together, and then the deep tissues and skin were reapproximated.

Pathology

**Figure 7:**
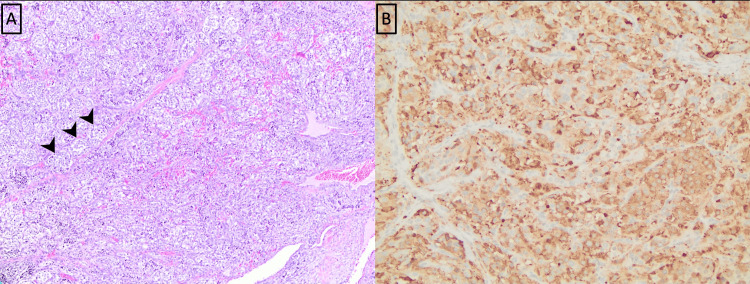
Microscopic evaluation of supraglottic paraganglioma. (A) Hematoxylin and eosin stain (200× magnification) shows clusters of cells (arrowhead on a few characteristic clusters) surrounded by thin vascular channels (zellballen pattern). (B) Chromogranin immunostain (200× magnification) is positive in the cytoplasm of tumor cells (brown pigment).

The left laryngeal mass consisted of an unoriented dark brown, slightly ragged mass measuring 3.1 × 2.9 × 2.0 cm. The mass was sectioned to reveal a solid tan-brown, variegated cut surface. The histology was similar to the thyroid paraganglioma with the classic histologic pattern of monomorphic cells arranged in nesting pattern with abundant sinusoidal capillaries between tumor nests. The tumor cells showed positive expression for synaptophysin and chromogranin, and S-100 highlighted the sustentacular cells (Figure 7). The neoplastic cells were positive for SDHB with no aberrant loss of stain.

## Discussion

Head and neck paragangliomas arise from the parasympathetic paraganglia in the neck and are therefore rarely functional (secrete catecholamines), in contradistinction to sympathetic origin paragangliomas in the chest and abdomen that more often secrete catecholamines [3]. Consequentially, clinical presentation of laryngeal and thyroid paragangliomas will most often be due to their mass effect on the airway (stridor) or as incidental findings on other imaging studies (incidental thyroid nodules) [3,4].

Paragangliomas in the head and neck are quite rare, accounting for 0.03% of all tumors and 0.6% of all head and neck tumors. Their estimated incidence is between 0.3 and 1 per 100,000 [7]. The majority originate from endocrine cell rests associated with the jugular bulb, glossopharyngeal nerve, vagus nerve, and carotid body [7]. In fact, 80% of these paragangliomas are associated with the carotid body or jugular bulb. Among these, vagus nerve-associated paragangliomas are exceptionally rare, with an incidence of 2.5% [8]. Laryngeal paragangliomas are even more uncommon within this category. Although definitive data is lacking, it is estimated that 90% of laryngeal paragangliomas are located in the supraglottic area, with 12% in the subglottic region, and 3% in the glottis [1]. Given their origin from the neural crest cells linked to the parasympathetic nervous system, they often associate with either the superior or recurrent laryngeal nerves [9]. Thyroid paragangliomas are also extremely rare, constituting fewer than 0.1% of all thyroid neoplasms and under 1% of all extra-adrenal paragangliomas [10,11].

As with paragangliomas of the head and neck in other locations, laryngeal and thyroid paragangliomas have a distinct female predominance [11]. A majority of paragangliomas arise sporadically, but a significant portion are linked to tumor syndromes such as multiple endocrine neoplasia type 2 (MEN2) and von Hippel-Lindau syndrome, among others [12]. Approximately 40% of cases show hereditary traits, often with mutations in the succinate dehydrogenase complex and related genes [13].

Imaging

In the thyroid, ultrasound often shows solid hypoechoic masses with increased vascularity, making them frequently indistinguishable from other solid hypervascular thyroid tumors such as papillary carcinoma [10,11]. Thyroid scintigraphy predominantly reveals “cold” nodules, though “hot” nodules have also been noted, making this modality less useful for narrowing the differential diagnosis [10,14,15]. Contrast-enhanced CT of the neck with contrast is useful for suggesting this diagnosis when surrounding abnormal vascularity is present, as in our cases. MRI is advantageous in detecting small (<5 mm) tumors or screening concurrent paragangliomas. T1 MRI with contrast is a preferred MRI sequence for laryngeal paragangliomas because the tumor typically presents with avid contrast enhancement. MRA or catheter angiogram will show the arterial supply of the paragangliomas, excluding a low-flow venous malformation [9]. Nuclear imaging with gallium or copper DOTATATE has great sensitivity for head and neck paragangliomas and may be used for preoperative evaluation, surveillance imaging, and screening for additional paragangliomas [16].

Histopathological evaluation

Differentiating paragangliomas from other pathologies hinges on a combination of histological, immunohistochemical, and clinical features. Carcinoid tumors, malignant melanomas, medullary thyroid carcinomas, and small-cell neuroendocrine carcinomas can all mimic laryngeal paragangliomas microscopically. Paragangliomas predominantly consist of chief and sustentacular cells [4]. The chief cells exhibit characteristic uniform polygonal nuclei and a pale eosinophilic cytoplasm, often arranged in a zellballen pattern [12]. Sustentacular cells with spindle-shaped nuclei appear around the tumor’s periphery. Chief cells are typically stained for chromogranin, synaptophysin, neuron-specific enolase, and CD56, while S-100 and SOX10 stains are indicative of the sustentacular cells [17]. Typical and atypical carcinoid tumors can histologically mimic a paraganglioma; however, a positive keratin expression and lack of S-100 expression helps to exclude a carcinoid tumor [18]. Additionally, the sustentacular cells’ immunoreactivity to S-100 aids in distinguishing the tumor from melanomas.

Owing to their histological resemblances, thyroid paragangliomas can be confused with other thyroid and extrathyroid tumors such as medullary thyroid carcinoma (MTC), hyalinizing trabecular tumor, and parathyroid adenoma [19]. Given their rarity, thyroid paragangliomas may be difficult to differentiate from other thyroid diseases solely by fine-needle aspiration (FNA) alone [10]. A 2020 single-center study investigated FNA results for five paragangliomas at their institution and found all of them to be misdiagnosed on FNA as follicular neoplasms. This group further went on to investigate the lesions for which frozen sections were obtained and found 3/3 paragangliomas to be incorrectly reported as follicular lesions, cellular lesions best deferred to permanent sections, and suspicious for MTC [20]. MTC poses a significant differential challenge. Both MTC and paragangliomas can consist of organoid cell clusters. Originating from the thyroid’s parafollicular C cells, MTC secretes calcitonin, and may have an elevated CEA, which can guide the diagnosis away from paraganglioma and toward MTC [19]. MTC commonly tests positive for stains such as cytokeratin, TTF-1, CEA, and calcitonin, while typically lacking the S-100 stain, given the absence of sustentacular cells [19]. Hyalinizing trabecular tumor, also known as paraganglioma-like adenoma, is another challenging thyroid tumor to differentiate from a thyroid paraganglioma. Negative postoperative immunohistochemical stains for TTF-1 and thyroglobulin can help distinguish it from paragangliomas [10]. Lastly, parathyroid adenoma, which can be found within the thyroid tissue, may also display positive staining for chromogranin A like paraganglioma. However, the presence of parathyroid hormone in immunostaining can help differentiate the two [10].

In exceedingly rare functional cases, FNA for these patients may be contraindicated without biochemical screening for catecholamine secretion or pre-FNA alpha-adrenergic blockade [20]. This is another avenue for imaging to guide clinical practice by suggesting the possibility of a thyroid paraganglioma on imaging before FNA to initiate serum catecholamine testing.

Molecular genetics

While many paragangliomas emerge sporadically, a significant portion is associated with various tumor syndromes, including succinate dehydrogenase-deficient pheochromocytoma-paraganglioma syndromes, MEN2, von Hippel-Lindau syndrome, neurofibromatosis 1, and Carney Stratakis syndrome [12,16]. Around 40% of cases are hereditary, showing an autosomal dominant pattern. Evaluation for mutations in tumor suppressor genes such as the succinate dehydrogenase complex (A, B, C, D), *SDHAF2 *gene, and SDHA-specific flavination protein (SDH-B and SDH-C being most common) is essential as these mutations increase the risk of multifocal disease and some portend an increased metastatic potential [9,13]. Mutations to the tumor suppressor genes *SHDB*, *SDHC*, and *SHDD* have been identified to heighten the risk of malignancy in paragangliomas over a patient’s lifetime, with the greatest risk of malignancy in patients exhibiting SDHB mutations [9,13].

Treatment and management

The mainstay of treatment for thyroid and laryngeal paragangliomas is surgical excision due to their radioresistant nature, with the extent of surgery being determined by tumor size and potential for malignancy [11]. Procedures such as lateral pharyngotomy are preferred for laryngeal tumors, especially for vascular tumors, owing to the potential bleeding risks associated with transoral resections. In addition, it is reported that the endoscopic transoral approach has an extremely high recurrence rate of 80% versus the 17% recurrence of other surgical approaches [21]. Complete excision while preserving the surrounding structures is essential, primarily focusing on maintaining voice and swallowing functions [22].

Preoperative embolization is often performed to reduce intraoperative blood loss. Some experts recommend selective embolization in tumors >2.5 cm in size [9,23]. While the ascending pharyngeal artery is often the primary feeding vessel in more typical paragangliomas such as carotid body and vagale paragangliomas, laryngeal and thyroid paragangliomas are more often supplied by the superior and inferior thyroidal arteries and their branches [1,24]. There is variability in the vascular supply to the thyroid and larynx in the degree of supply from the superior versus inferior thyroidal arteries, and the inferior thyroidal arteries may arise from either the thyrocervical trunk or the subclavian artery. Knowledge of these anatomical variations is essential when performing selective angiography for preoperative embolization.

The long-term prognosis for these predominantly benign tumors is generally favorable. However, due to the uncertain biological behavior and, albeit rare, potential for malignancy, diligent imaging, and clinical monitoring are essential, particularly in tumors with higher-risk genetic profiles [1,10]. Chemotherapy may be recommended for unresectable or recurrent tumors [1].

## Conclusions

In summary, the diagnosis and management of paragangliomas originating in the laryngeal paraganglia, whether thyroid or laryngeal, demands a multidisciplinary approach. This involves advanced imaging, meticulous histopathological examination, and an understanding of molecular genetics. Proper differentiation from other pathologies ensures accurate treatment, which, when combined with diligent follow-up, can result in favorable patient outcomes.
